# Transcriptome analysis in contrasting maize inbred lines and functional analysis of five maize NAC genes under drought stress treatment

**DOI:** 10.3389/fpls.2022.1097719

**Published:** 2023-01-19

**Authors:** Ning Ding, Ying Zhao, Weixiang Wang, Xuyang Liu, Wentong Shi, Dengfeng Zhang, Jiajie Chen, Shuo Ma, Qingpeng Sun, Tianyu Wang, Min Lu

**Affiliations:** ^1^ Beijing Key Laboratory for Agricultural Application and New Technique, College of Plant Science and Technology, Beijing University of Agriculture, Beijing, China; ^2^ Institute of Crop Science, Chinese Academy of Agricultural Sciences/the National Key Facility for Crop Gene Resources and Genetic Improvement (NFCRI), Beijing, China

**Keywords:** maize, drought stress, NAC, transcriptome analysis, transgenic *Arabidopsis*

## Abstract

Drought substantially influences crop growth and development. NAC (NAM, ATAF1/2, and CUC2) transcription factors (TFs) have received much attention for their critical roles in drought stress responses. To explore the maize NAC genes in response to drought stress, the transcriptome sequencing data of NAC TFs in two maize inbred lines, the drought tolerance line H082183 and the sensitive line Lv28, were used to screen the differentially expressed genes (DEGs). There were 129 maize NAC protein-coding genes identified, of which 15 and 20 NAC genes were differentially expressed between the two genotypes under MD and SD treatments, respectively. Meanwhile, the phylogenetic relationship of 152 non-redundant NAC family TFs in maize was generated. The maize NAC family proteins were grouped into 13 distinct subfamilies. Five drought stress–responsive NAC family members, which were designed as ZmNAP, ZmNAC19, ZmNAC4, ZmJUB1(JUBGBRUNNEN1), and ZmNAC87, were selected for further study. The expression of *ZmNAP*, *ZmNAC19*, *ZmNAC4*, *ZmJUB1*, and *ZmNAC87* were significantly induced by drought, dehydration, polyethylene glycol (PEG) stress, and abscisic acid (ABA) treatments. The overexpressing *Arabidopsis* of these five NAC genes was generated for functional characterization, respectively. Under different concentrations of NaCl, D-mannitol stress, and ABA treatments, the sensitivity of *ZmNAP-*, *ZmNAC19-*, *ZmNAC4-*, *ZmJUB1-*, and *ZmNAC87-*overexpressing lines was significantly increased at the germination stage compared to the wild-type lines. The overexpression of these five NAC members significantly improved the drought stress tolerance in transgenic *Arabidopsis*. Yeast two-hybrid screening analysis revealed that ZmNAP may cooperatively interact with 11 proteins including ZmNAC19 to activate the drought stress response. The above results inferred that *ZmNAP*, *ZmNAC19*, *ZmNAC4*, *ZmJUB1*, and *ZmNAC87* may play important roles in the plant response to drought stress and may be useful in bioengineering breeding and drought tolerance improvement.

## Introduction

Maize (*Zea mays* L.) is an important crop, which is widely grown as food, feed, and energy in the world. However, abiotic stresses, especially drought stresses, severely affected maize growth and yield. Therefore, mining and characterizing drought-responsive genes in maize would benefit maize breeding, reducing maize yield loss in arid areas.

Abiotic stresses seriously influence plant growth and development, causing reactive oxygen species (ROS) accumulation, stomatal closure, photosynthesis, and crop yield reduction (Gong et al., 2020). The plant has evolved complex mechanisms at physiological and biochemical levels to respond to abiotic stresses (Zhang et al., 2022). Over the past decades, a series of stress-responsive genes had been characterized in plants (Fan et al., 2018). NAC (NAM, ATAF1/2, and CUC2) proteins are plant-specific transcriptional regulators that constitute a large gene family and play vital roles in lateral root development, secondary cell wall biosynthesis, xylem development, leaf senescence, and abiotic stress resistance (Balazadeh et al., 2010; Ohashi-Ito et al., 2010; Nuoendagula et al., 2018). NAC proteins are typically characterized by a highly conserved DNA-binding domain at its N-terminus, which was recognized as the NAC domain. The NAC domain could be divided into five subdomains A, B, C, D, and E. The divergent C-terminal region was considered as the transcriptional activation domain (Tran et al., 2004). Recently, NAC proteins have received wide attention from researchers for their vital roles in the plant response to drought stress.

In recent years, a variety of abiotic stress-related NAC TFs have been identified. In *Arabidopsis*, the overexpression of *ANAC019*, *ANAC055*, or *ANAC072* significantly improved drought stress tolerance (Tran et al., 2004). In rice, SNAC1, OsNAC5, and OsNAC10 improved the drought and salt stress responses of rice *via* an abscisic acid (ABA)–dependent pathway (Hu et al., 2006; Jeong et al., 2010; Takasaki et al., 2010). Rice ONAC066 activated the transcription of *OsDREB2A* by directly binding to a AtJUB1 binding site (JBS)-like *cis*-element in the promoter region, improving drought and oxidative stress responses (Yuan et al., 2019). In maize, the overexpression of *ZmNAC111* in maize significantly increased water use efficiency by upregulating the expression of drought stress–responsive genes, thereby enhancing drought resistance (Mao et al., 2015). *ZmSNAC13-* overexpressing *Arabidopsis* performed enhanced drought stress tolerance *via* promoting the expression of *PYL9* and *DREB3* (Luo et al., 2022). Maize ZmNAC48, which interacted with *cis*-NATZmNAC48 through an siRNA-dependent mechanism, was involved in drought stress responses by regulating stomatal closure (Mao et al., 2021). Our previous study indicated that the overexpression of *ZmSNAC1* significantly improved the dehydration tolerance in transgenic *Arabidopsis* (Lu et al., 2012). To date, only a few studies of maize NAC transcription factors (TFs) that respond to drought stress have been identified.

The maize drought–tolerant inbred lines H082183 (H) and drought-sensitive inbred lines Lv28 (L) were used in this study. Under drought stress conditions, the leaf-relative water contents of H082183 were significantly higher than those of Lv28 after a 9–44-day drought (Zhang et al., 2017). We analyzed the transcriptome data of the two genotypes under drought stress and control treatments, and five drought-responsive maize NAC genes *ZmNAP, ZmNAC19, ZmNAC4, ZmJUB1*, and *ZmNAC87* were characterized. Functional characterization indicated that the overexpression of these five maize NAC family genes performed significantly enhanced drought stress tolerance compared to the wild-type (WT) lines. Therefore, these results may lay the foundation for future studies to explore the detailed molecular mechanisms of maize NAC proteins in the drought stress response.

## Methods and materials

### Transcriptome analysis

To explore the expression level of NAC family TFs under drought stress treatment, the transcriptome sequencing data of NAC family TFs in two maize inbred lines: the tolerant line H082183 and the sensitive line Lv28 under well-watered conditions and natural soil drought stress were downloaded from the NCBI Sequence Read Archive (SRA, accession SRP102142). Transcriptional sequencing data were treated according to the method of Zhang et al., 2017. Clean reads were aligned to the maize reference genome B73_RefGen_v3 sequence by the Tophat2 software. The fragments per kilobase million mapped reads (FPKM) of each gene was calculated by Cuffdiff to estimate gene expression levels.

### Phylogenetic analysis

There were 189 maize NAC TF sequences obtained from the Plant Transcription Factor Database (http://planttfdb.cbi.pku.edu.cn/), and ClustalW2 was used for protein sequence alignment to remove redundant sequences. The phylogenetic tree of non-redundant NAC family TFs in maize were generated by PhyML version 2.4.3 using maximum likelihood methods. The phylogenetic tree was analyzed and displayed with MEGA7 software using the neighbor-joining method (Kumar et al., 2016).

### Plant materials and treatments

The seeds of the maize inbred line B73 were grown in plastic containers filled with a mixture of nutrient soil and vermiculite (1:1, v/v) and were watered suficiently before initiating the drought stress treatment experiments. The three-leaf stage maize seedlings were subjected to moderate and severe drought stress; then, water was resumed after severe drought stress. The soil moisture content and leaf relative water content were measured during drought stress. The shoots were harvested under moderate stress, severe stress, and rewatering treatments with the leaf-relative water content of 45%, 33%, and 96%, respectively, to analyze the expression patterns of NAC genes under drought stress and rewatering treatments. For the dehydration treatment, the seedlings were dried in air on Whatman filter papers. The shoots and roots were harvested at given time points (0, 1, 3, 6, 12, and 24 h) and frozen in liquid nitrogen immediately.

The seeds of a drought-sensitive inbred line Lv28 and a drought-tolerant inbred line H082183 were provided by the Institute of Crop Sciences, Chinese Academy of Agricultural Sciences, and preserved in our laboratory. The seeds of these two maize inbred lines were sown in the soil, and the seedlings at the three-leaf stage were used for soil drought stress, dehydration, and ABA and PEG treatments. For ABA and PEG treatments, the seedlings grown in vermiculite were rinsed and immersed into the Hoagland solution supplemented with 100 μM ABA, 20% (w/v) PEG, respectively. The Hoagland solution supplemented with nothing in it was treated as the control. The shoots and roots were harvested at given time points (0, 1, 3, 6, 12, and 24 h) and frozen in liquid nitrogen immediately.

### Real-time quantitative PCR analysis

Total RNAs were extracted from appropriate maize tissues using the TRIzol reagent according to the manufacturer’s specifications (TakaRa, China). The RNA samples were reverse-transcribed using the RevertAid First Strand complementary DNA (cDNA) Synthesis Kit (Thermo Scientific, USA). Gene-specific primers for maize NAC genes were designed with the Roche LCPDS2 software. The primer sequences used in the RT-qPCR are listed in **Supplementary Table 1**. RT-qPCR was performed using a LightCycler^®^ 480 II Real-time PCR Instrument (Roche, Swiss) with SYBR Premix Ex TaqTM II (TakaRa, China). Each sample was run in triplicate for analysis. The maize *GAPDH* (*NM_001111943*) gene was used for RT-qPCR as an internal control for normalization, and the 2^-ΔΔCt^ method was used to calculate the relative expressions of NAC genes under different stress treatments.

### Transactivation activity assay

The full-length coding sequences (CDSs) and the truncated N- and C-terminal regions of *ZmNAP*, *ZmNAC19*, *ZmNAC4*, *ZmJUB1*, and *ZmNAC87* were amplified from the B73 maize inbred line by PCR and connected to the pGBKT7 vector, respectively. The constructs and the negative-control pGBKT7 were transformed into the yeast strain AH109. The transformants were spread on SD/-Trp, SD/-Trp/-His, and SD/-Trp/-His supplemented with 10, 20, and 30 mM 3-AT media to detect the transactivation activity.

### Generation of overexpressing transgenic *Arabidopsis*

The full-length CDSs of *ZmNAP*, *ZmNAC19*, *ZmNAC4*, *ZmJUB1*, and *ZmNAC87* were cloned into the pCAMBIA3301 vector and transferred into *Arabidopsis* using the floral dip method *via Agrobacterium tumefaciens* GV3101 (Clough and Bent, 1998). The seeds of the positive transgenic plants were screened on the MS agar medium containing 7.5 mg/L *phosphinothricin* (PPT) after vernalization, and the seedlings were transplanted into the soil. The genomic DNA for each plant was extracted, and the bar and target genes were detected with PCR. Positive transgenic plants were screened according to a segregation ratio (resistant/sensitive = 3:1) and confirmed by PCR. Homozygote T3 generation plants, which are 100% resistant to PPT, were used for phenotype analysis. The RT-qPCR method was used to evaluate the transcripts of *ZmNAP*, *ZmNAC19*, *ZmNAC4*, *ZmJUB1*, and *ZmNAC87* in overexpressing *Arabidopsis.* The expression of the *Actin* gene (*NM_112764*) of *Arabidopsis* was used as an internal control. The relative expression levels were calculated using the relative 2^-ΔΔCt^ method (Livak and Schmittgen, 2001). Three biological replicated assays were carried out for each experiment.

### Phenotype analysis of *ZmNAP-*, *ZmNAC19-*, *ZmNAC4-*, *ZmJUB1-*, and *ZmNAC87-*overexpressing *Arabidopsis*

The seeds of each homozygous plant were sterilized and sown on an MS agar medium containing 400 mM D-Mannitol, 175 mM NaCl, and 1.5 μM ABA, respectively. The seeds were grown in a greenhouse (21°C; humidity 40%–50%; 16-h light/8-h darkness). The germination ratio was calculated daily for seven consecutive days.

Seeds of the WT and each homozygous plant were surface-sterilized with 75% (w/v) sodium hypochlorite and grown on MS media for approximately 6 days. Then, the WT and transgenic plants were transferred into the soil and grown under normal irrigation conditions for 3 weeks, the relative humidity was 20%–30%, and the temperature was 21°C. For drought stress treatment, the plants were withheld water for 2 weeks. The phenotype of the WT and transgenic plants under the drought stress treatment was measured, and the survival rate was calculated after 1 week of rewatering. Each experiment was repeated at least three times independently, and the average and standard errors were calculated.

### Yeast two-hybrid screening

To screen target proteins that interacted with ZmNAP, the different truncated CDSs (1-186aa, 1-207aa, and 1-227aa) of ZmNAP were amplified and ligated into the pGBKT7 vector and transformed into Y2HGold yeast. The pGBKT7 vector was used as the negative control. All transformants were grown in SD/-Trp and SD/-Trp/X-α-Gal media to identify the self-activating activity as the bait. In yeast two-hybrid screening, the bait plasmid and the maize cDNA library plasmids were cotransformed into Y2HGold yeast. The transformants were spread and selected on the SD/-Ade/-His/-Leu/-Trp/X-α-Gal/AbA medium. The target prey plasmids were isolated using the Easy Yeast Plasmid Isolation Kit (Tiangen, Beijing, China) and interacted with a bait plasmid again in Y2HGold yeast.

## Results

### Identification and phylogenetic analysis of NAC family TFs in maize

**Figure 1:**
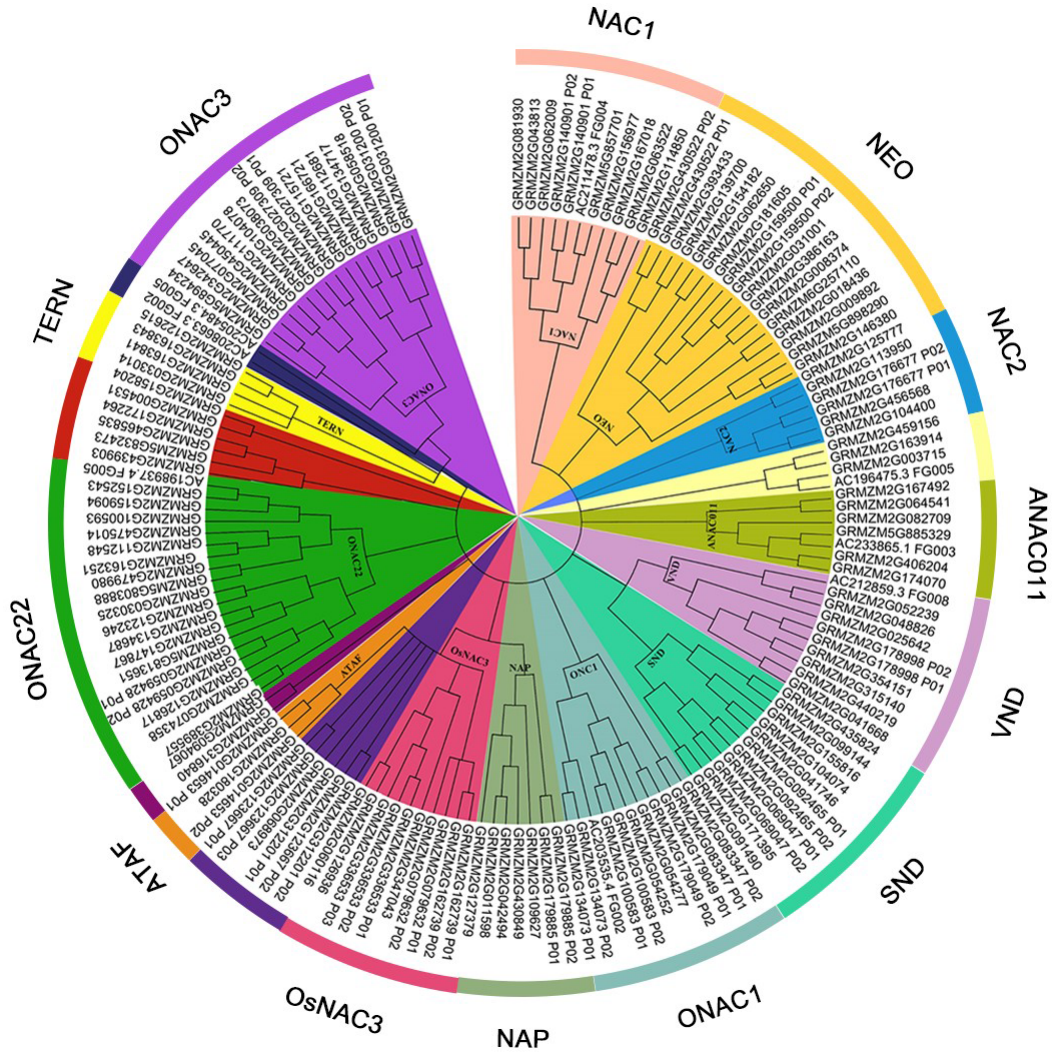
Neighbor-joining tree of NAC (NAM, ATAF1/2, and CUC2) family transcription factors (TFs) in maize. The bootstrap values (1,000 replications) are presented on the relevant nodes.

To identify the NAC TF in maize, the 189 NAC protein sequence of maize was obtained from the Plant Transcription Factor Database. After removing the redundant sequence, 152 non-redundant NAC protein sequences containing 129 maize NAC protein sequences mentioned in the transcription data were obtained. Detailed information including the gene IDs of maize NAC genes are shown in **Supplementary Table 2**. The evolution analysis of 152 non-redundant NAC proteins was performed. As shown in **Figure 1**, the maize NAC family proteins were grouped into 13 distinct subfamilies, which were ONAC3, TERN, ONAC22, ATAF, OsNAC3, NAP, ONAC1, SNO, VNO, ANAC011, NAC2, NEO, and NAC1 subfamilies.

### Differential expression analysis of maize NAC genes under field drought conditions

**Figure 2:**
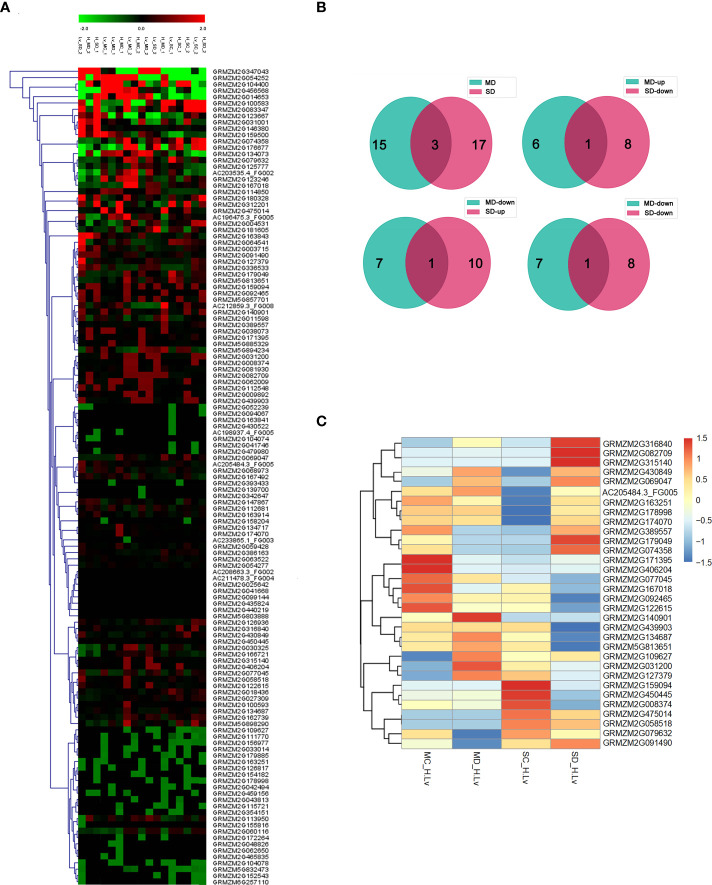
Differential expression analysis of maize NAC genes. **(A)** Heat map analysis of the NAC TF expression under natural soil drought treatments in H082183 and Lv28 based on RNA-seq experiments. Red represents high expression, and green represents low expression. MD, SD, MC, and SC stand for moderate drought, severe drought, moderate drought control, and severe drought control, respectively. **(B)** Numbers of drought-responsive differentially expressed genes (DEGs) in H082183, compared to Lv28 under the drought stress treatment. MD, moderate drought stress treatment; SD, severe drought stress treatment. **(C)** Hierarchical clustering based on log2 (fold changeH082183/fold changeLv28) values of DEGs.

To explore the response of maize NAC gene to drought stress, we downloaded the genome-wide expression data of two maize inbred lines: the tolerant line H082183 and the sensitive line Lv28 under well-watered conditions and soil drought stress. There were 129 NAC protein-coding genes obtained (**Supplementary Table 3**). The results of transcriptional analysis showed that 28 and 39 NAC genes were specifically upregulated; 21 and 27 NAC genes were uniquely downregulated in H082183 and Lv28 under at least one drought stress condition. There were 32 NAC genes (15 genes in MD, 20 genes in SD, and 3 genes in both MD and SD) differentially expressed in the two genotypes under at least one drought stress condition (|log_2_(fold change)| > 1, FDR < 0.05), respectively (**Figure 2**).

### Transcriptional analysis of selected NAC genes involved in drought and dehydration conditions

**Figure 3:**
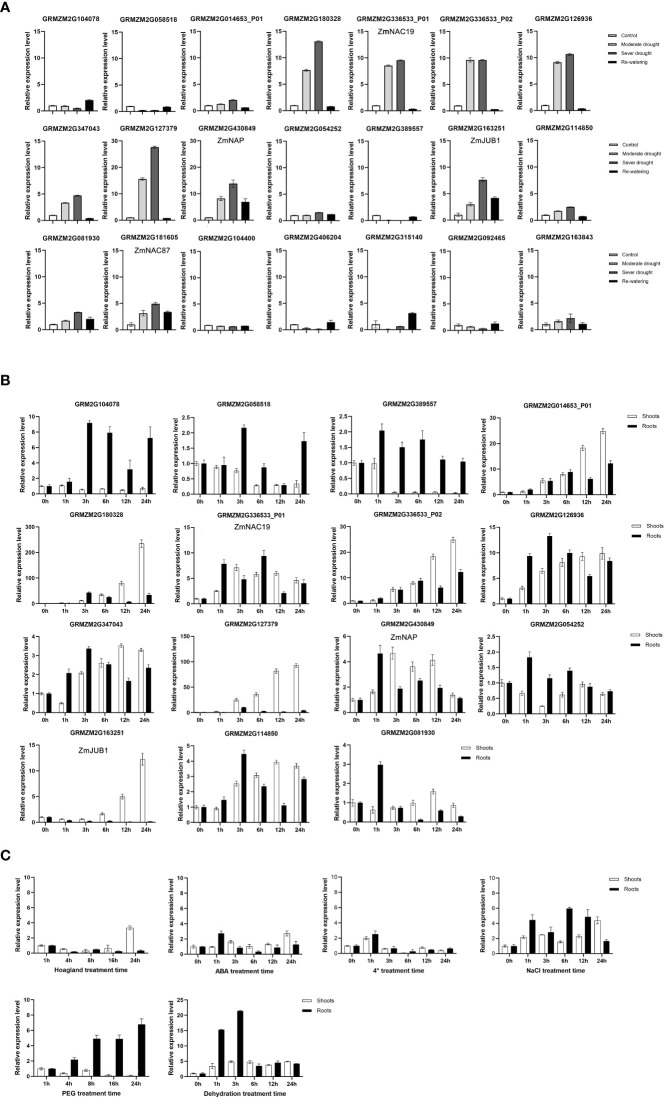
Expression levels analysis of maize NAC genes. **(A)** Expression analysis of 21 maize NAC genes under drought stress treatments. **(B)** Expression analysis of 15 maize NAC genes under dehydration treatments. Maize ZmGAPDH (NM_001111943) was used as the internal reference gene. **(C)** Expression patterns of *ZmNAP* in shoots and roots when treated with 200 mM NaCl, 100 μM abscisic acid (ABA), 20% PEG, dehydration, low temperature (4°C), drought stress, and control (Hoagland), respectively. The maize *ZmGAPDH* (*NM_00111943*) gene was used as an internal control for normalization.

Combined with the results of RNA-Seq analysis and phylogenetic analysis, 21 NAC genes from 13 subgroups were selected for further study. To determine the effect of drought on the expression of selected NAC genes, RT-qPCR was used to identify temporal expression patterns at the seedling stage (**Figure 3A**). The results showed that the expression levels of nine NAC genes (*GRMZM2G163251*, *GRMZM2G181605*, *GRMZM2G347043*, *GRMZM2G180328*, *GRMZM2G430849*, *GRMZM2G336533_P01*, *GRMZM2G126936*, *GRMZM2G336533_P02*, and *GRMZM2G127379*) were significantly increased under drought stress treatment (compared with normal watering, the expression fold ≥ 2 under MD and SD treatments). These NAC genes were induced by drought stress; the results were consistent with transcriptome analysis in the two genotypes.

Moreover, in the dehydration assays, the expression levels of 15 NAC genes in shoots and roots were detected after 0–24 h dehydration treatment (**Figure 3B**). Among them, *GRMZM2G180328*, *GRMZM2G127379*, and *GRMZM2G163251* were induced mainly in shoots, whereas *GRMZM2G104078* and *GRMZM2G389557* were induced mainly in roots. *GRMZM2G014653-P01*, *GRMZM2G336533_P01*, *GRMZM2G336533_P02*, *GRMZM2G126936*, *GRMZM2G347043*, *GRMZM2G430849*, and *GRMZM2G114850* were induced both in shoots and roots.

### Five selected NAC genes were responsive to drought stress

Five drought-responsive maize NAC members (GRMZM2G430849, GRMZM2G336533_P01, GRMZM2G068973, GRMZM2G163251, and GRMZM2G181605) were selected, and the full-length CDSs were obtained from the maize inbred line B73, respectively. GRMZM2G430849, GRMZM2G336533_P01, GRMZM2G068973, GRMZM2G163251, and GRMZM2G181605 had the highest identity to ANAC047(AEE74033), ZmSNAC1(AEY78612), OsNAC4(BAB64820), JUB1(At2g43000), and ANAC087 (At5g18270) and were designed as *ZmNAP*, *ZmNAC19*, *ZmNAC4*, *ZmJUB1*, and *ZmNAC87*, respectively.

RT-qPCR showed that the expression levels of Z*mNAP*, *ZmNAC19*, *ZmJUB1*, and *ZmNAC87* were significantly increased under drought stress in B73 (**Figure 3**). We further detected the expression levels of these five genes in Lv28 and H082183 under drought treatment. The expression levels of *ZmNAC4* and *ZmJUB1* were significantly upregulated under drought treatment in Lv28, whereas there were no significant differences in H082183. The expression level of *ZmNAC19* was remarkably increased both in Lv28 and H082183 under drought treatment. Meanwhile, the expression level of *ZmNAC87* was significantly upregulated in Lv28 and downregulated in H082183 under the drought (**Supplementary Figure 1**). The results suggested that these five NAC genes may be involved in the drought stress response in maize.

### Overexpression of *ZmNAP*, *ZmNAC19*, *ZmNAC4*, *ZmJUB1*, and *ZmNAC87* improved sensitivity under ABA, high salt, and D-mannitol stress treatments at the germination stage

**Figure 4:**
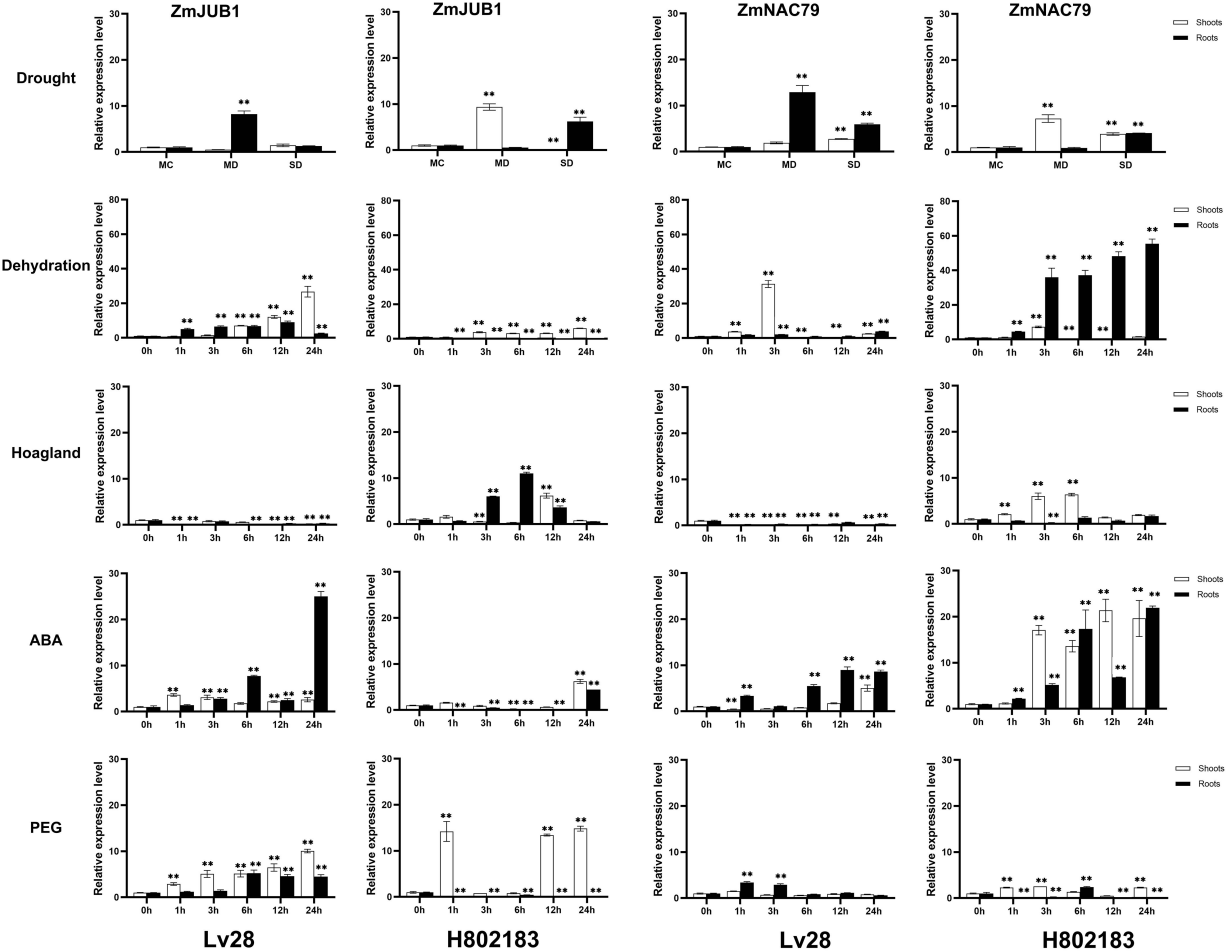
Expression analysis of ZmJUB1 and ZmNAC87 under drought stress, dehydration, Hoagland, 100 μM ABA, and 20% PEG treatments in Lv28 and H082183. Maize ZmGAPDH (NM_001111943) was used as the internal reference gene. Highly significant differences (P < 0.01) were represented by **.

ZmNAC19 and ZmNAC4 had been proven to be strongly induced by drought, NaCl, and ABA in maize in our previous study (Lu et al., 2015). In this study, RT-qPCR showed that, ZmNAP, ZmJUB1, and ZmNAC87 were induced by drought stress, dehydration, and PEG and exogenous ABA treatments (**Figures 3C**, **4**). To functionally characterize maize ZmNAP, ZmNAC19, ZmNAC4, ZmJUB1, and ZmNAC87 under drought stress conditions, *ZmNAP-*, *ZmNAC19-*, *ZmNAC4-*, *ZmJUB1-*, and *ZmNAC87-* overexpressing *Arabidopsis* were generated, respectively. The relative expression levels of transgenic lines were evaluated, and the transgenic lines of overexpressed *ZmNAP* (OX-47, OX-21), *ZmNAC19* (OX-15, OX-17, OX-23), ZmNAC4 (OX-22, OX-14), *ZmJUB1* (OX-3, OX-7, OX-9, and OX-44), and *ZmNAC87* (OX-58 and OX-60) were used for phenotypic analysis, respectively.

**Figure 5:**
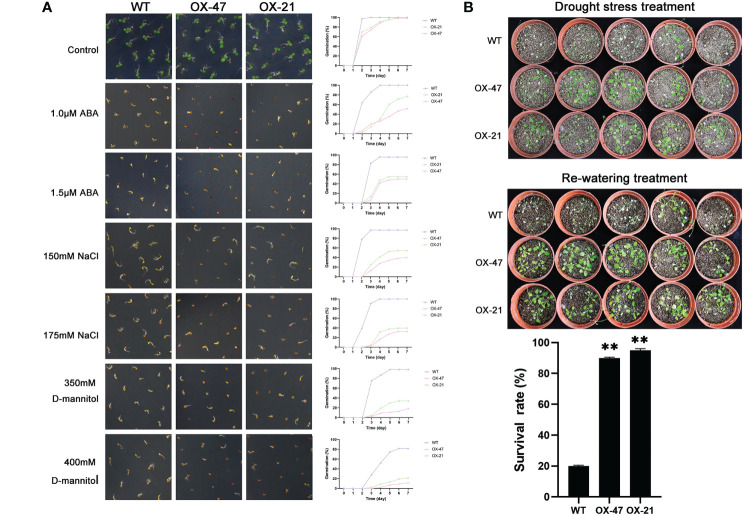
Germination and phenotype analysis ZmNAP overexpressing Arabidopsis. **(A)** Germination assays of ZmNAP were performed under the presence of 1.0 μM and 1.5 μM ABA, 150 mM and 175 mM NaCl, 350 mM and 400 mM D-mannitol. Data are means ±SD of three biological replicates. **(B)** Performance and survival rate of the ZmNAP overexpressing lines under drought stress and re-watering treatments. Three biological replicates and calculate the average. Highly significant differences (P < 0.01) were represented by **.

**Figure 6:**
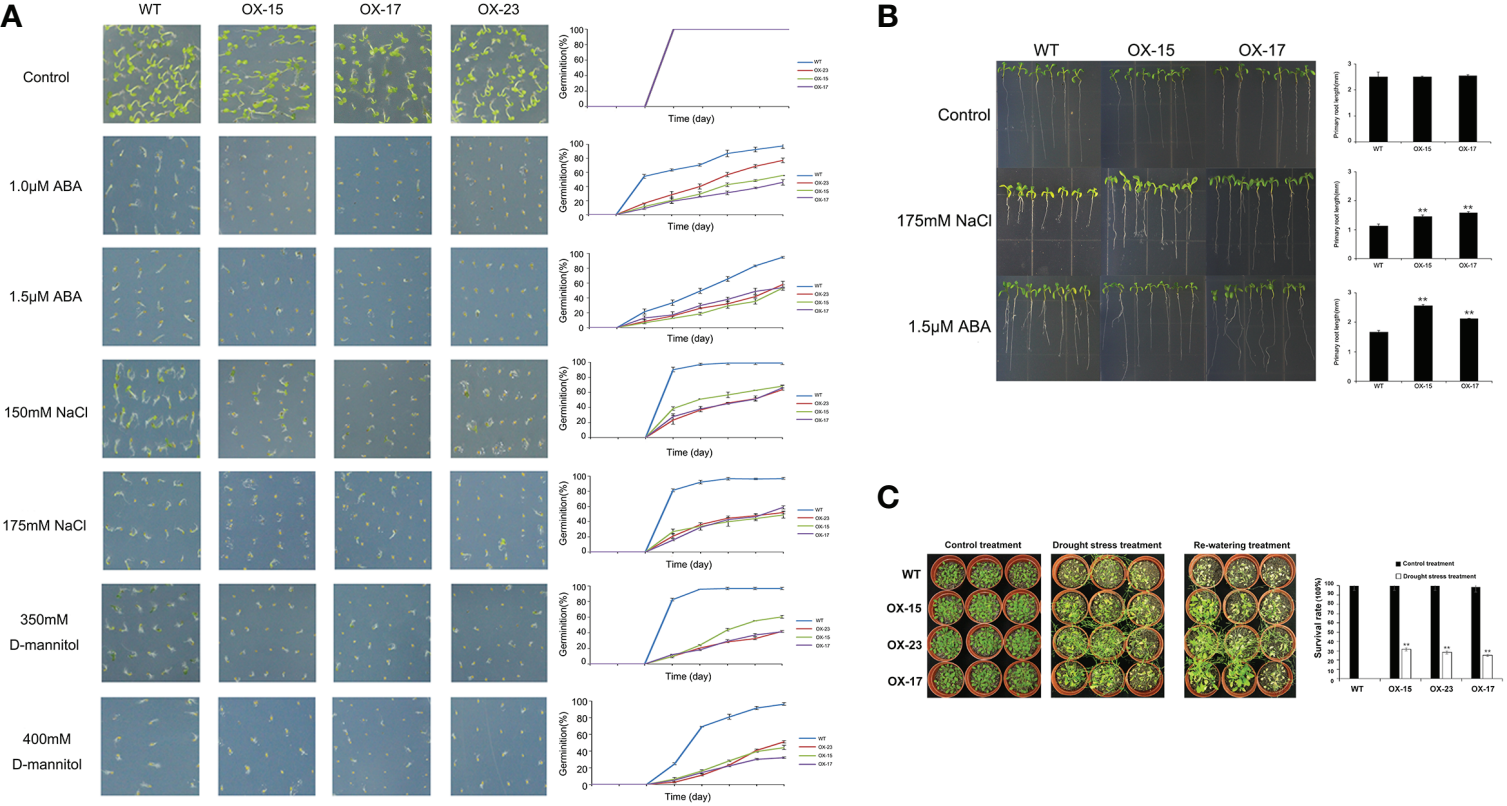
Germination and phenotype analysis ZmNAC19 overexpressing Arabidopsis. **(A)** Germination assays of ZmNAC19 were performed under the presence of 1.0 μM and 1.5 μM ABA, 150 mM and 175 mM NaCl, 350 mM and 400 mM D-mannitol. Each measurement consists of 50 seeds. Data are means ±SD of three biological replicates for 8 days. **(B)** Root length of the ZmNAC19 overexpressed transgenic lines in the presence of 1.5 μM ABA and 175 mM NaCl. **(C)** Performance and survival rate of the ZmNAC19 overexpressing lines under drought stress and re-watering treatments. Three biological replicates and calculate the average. Highly significant differences (P < 0.01) were represented by **.

**Figure 7:**
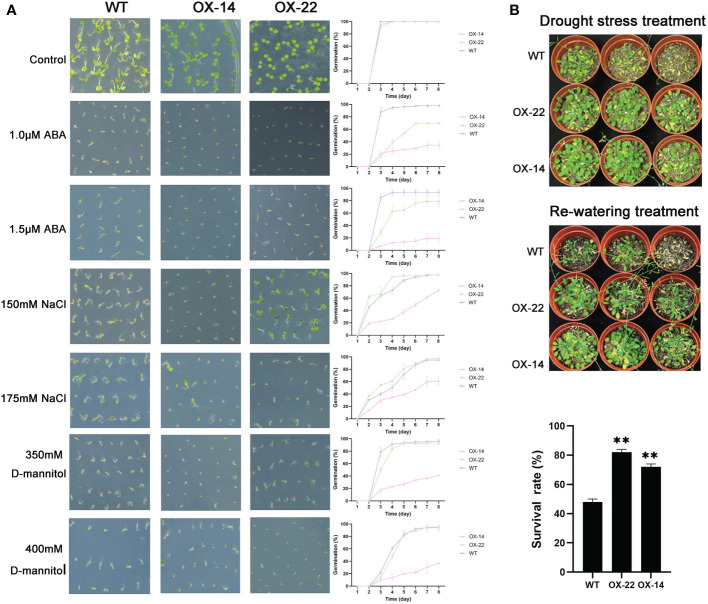
Germination and phenotype analysis ZmNAC4 overexpressing Arabidopsis. **(A)** Germination assays of ZmNAC4 were performed under the presence of 1.0 μM and 1.5 μM ABA, 150 mM and 175 mM NaCl, 350 mM and 400 mM D-mannitol. Each measurement consists of 50 seeds. **(B)** Performance and survival rate of the ZmNAC4 overexpressing lines under drought stress and re-watering treatments. Three biological replicates and calculate the average. Highly significant differences (P < 0.01) were represented by **.

**Figure 8:**
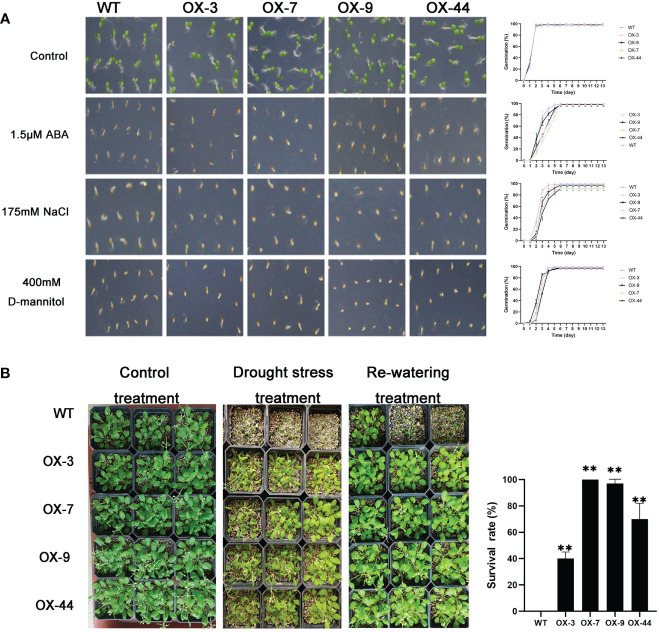
Germination and phenotype analysis ZmJUB1 overexpressing Arabidopsis. **(A)** Germination assays of ZmJUB1 were performed under the presence of 1.5 μM ABA and 175 mM NaCl and 400 mM D-mannitol. Each measurement consists of 50 seeds. Data are means ±SD of three biological replicates for 13 days. **(B)** Performance and survival rate of the ZmJUB1 overexpressing lines under drought stress and re-watering treatments. Three biological replicates and calculate the average. Highly significant differences (P < 0.01) were represented by **.

**Figure 9:**
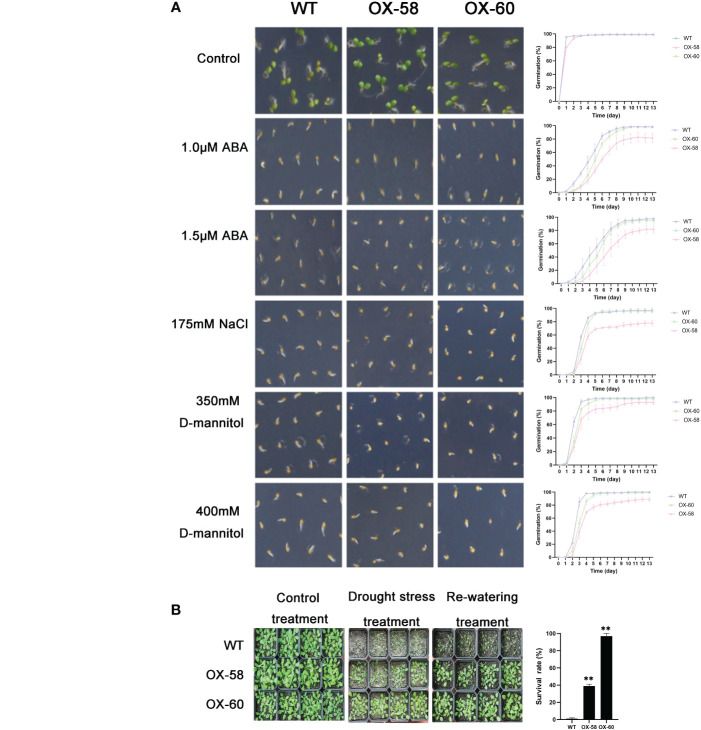
Germination and phenotype analysis *ZmNAC87* overexpressing *Arabidopsis*. **(A)** Germination assays of *ZmNAC87* were performed under the presence of 1.0 μM ABA, 1.5 μM ABA, 150 mM NaCl, 175 mM NaCl, 350 mM D-mannitol and 400 mM D-mannitol. Each measurement consists of 50 seeds. Data are means ±SD of three biological replicates for 13 days. **(B)** Performance and survival rate of the *ZmNAC87* overexpressing lines under drought stress and re-watering treatments. Three biological replicates and calculate the average. Highly significant differences (P < 0.01) were represented by **.

Compared to the WT, *ZmNAP-*, *ZmNAC19-*, *ZmNAC4-*, *ZmJUB1-*, and *ZmNAC87*-overexpressing lines exhibited significantly enhanced sensitivity to ABA, salt, and D-mannitol at the germination stage (**Figures 5A**, **6A**, **7A**, **8A**, **9A**), whereas no significant differences could be observed under normal conditions. Under 175 mM NaCl treatment, compared to the WT lines, the germination rates of *ZmNAP-*, *ZmNAC19-*, *ZmNAC4-*, *ZmJUB1*, and *ZmNAC87-*overexpressing lines decreased by 53.1%–65.5%, 24%–44%, 40%–60%, 15%–40%, and 18%–32%, respectively. Similarly, under 1.5 μM ABA stress treatment, the germination rates of transgenic lines decreased by 40%–48.6%, 32%–52%, 23%–43%, 9%–42%, and 6%–30%, respectively. It may be inferred that the *ZmNAP*, *ZmNAC19*, *ZmNAC4*, *ZmJUB1*, and *ZmNAC87* may respond to drought stress *via* ABA-dependent signaling pathways.

### Overexpression of *ZmNAP, ZmNAC19, ZmNAC4, ZmJUB1, and ZmNAC87* enhanced drought stress tolerance in transgenic *Arabidopsis*

Drought tolerance assays were further performed to explore the biological functions of ZmNAP, ZmNAC19, ZmNAC4, ZmJUB1, and ZmNAC87. The results indicated that, compared with the WT, the survival rate of transgenic plants improved under drought stress conditions (**Figures 5B**, **6C**, **7B**, **8B**, **9B**). Furthermore, the survival rate of *ZmNAP-*, *ZmNAC19-*, *ZmNAC4-*, *ZmJUB1-*, and *ZmNAC87-* overexpressing lines improved by 65%–67%, 30%–35%, 24%–35%, 40%–97%, and 35%–95% compared with the WT, respectively. We concluded that overexpression of *ZmNAP*, *ZmNAC19*, *ZmNAC4*, *ZmJUB1*, and *ZmNAC87* could significantly increase transgenic plants’ drought stress tolerance. Moreover, the overexpression of *ZmNAC19* significantly improved the primary root length of transgenic *Arabidopsis* under NaCl and ABA treatments (**Figure 6B**).

### ZmNAP, ZmNAC19, ZmNAC4, ZmJUB1, and ZmNAC87 showed transactivation activity

**Figure 10:**
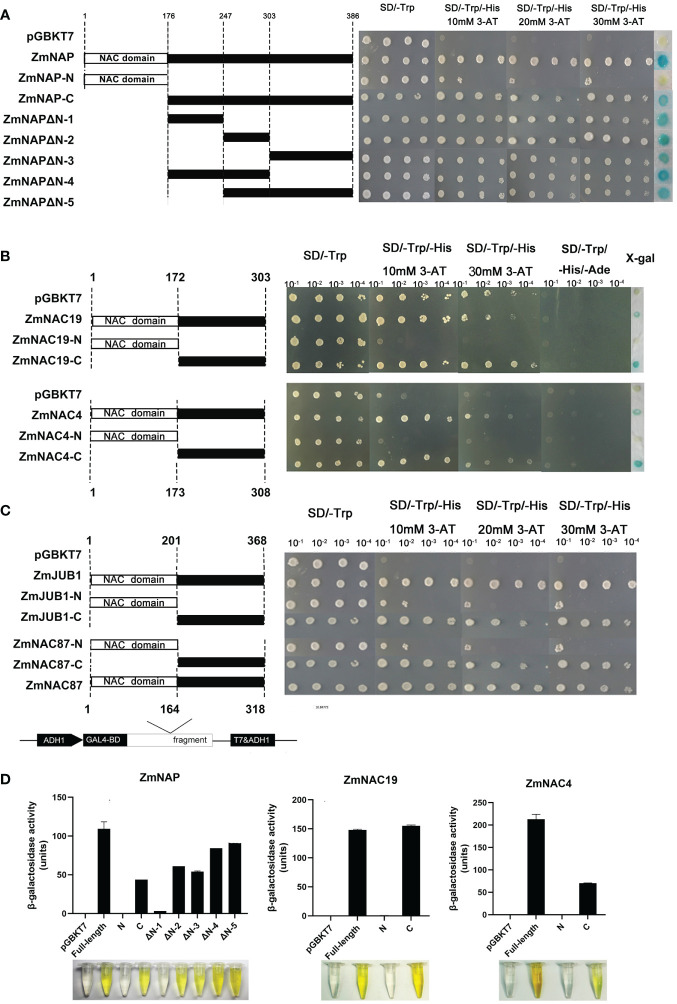
Transactivation analysis of the full-length CDSs and different truncations of ZmNAP, ZmNAC19, ZmNAC4, ZmJUB1, and ZmNAC87. **(A)** Transactivation activity analysis of the full-length CDSs and different truncations of ZmNAP. The Recombinant plasmids pGBKT7-ZmNAP-C, pGBKT7-ZmNAP-N, pGBKT7-ZmNAPΔN-1, pGBKT7-ZmNAPΔN-2, pGBKT7-ZmNAPΔN-3, pGBKT7- ZmNAPΔN-4, pGBKT7- ZmNAPΔN-5 and prey plasmid pGBKT7 were transformed into the yeast strain AH109 and selected on the SD/-His/-Trp/3-AT medium. **(B)** Transactivation activity analysis of the full-length CDSs and different truncations of ZmNAC19 and ZmNAC4. The yeast transformation and screening methods are described above. **(C)** Transactivation activity analysis of the full-length CDSs and different truncations of ZmJUB1 and ZmNAC87. The yeast transformation and screening methods are described above. **(D)** β-galactosidase activity of *ZmNAP*, *ZmNAC19*, and *ZmNAC4* were determined in yeast strain Y187. The pGBKT7 vector was used as a negative control.

To examine the transactivation activity of the ZmNAP, ZmNAC19, ZmNAC4, ZmJUB1, and ZmNAC87, the constructs that recombinant pGBKT7 and the different truncations of these five NAC genes were transformed into the yeast strain AH109, respectively. The transformants of pGBKT7 connection with the full-length and C-terminal region of these five genes grew well on the SD/-Trp and SD/-Trp/-His/30 mM 3-AT media, whereas the control (pGBKT7 vector) and pGBKT7 connection with the N-terminal region of these five genes’ transformants failed to grow. The results indicated that the full-length and C-terminus of ZmNAP, ZmNAC19, ZmNAC4, ZmJUB1, and ZmNAC87 had a transactivation activity in yeast. ONPG assays further confirmed that the full-length and C-terminus of ZmNAP, ZmNAC19 and ZmNAC4 have strong transactivation activity (**Figure 10**).

### ZmNAP interacted with 11 proteins in yeast

**Figure 11:**
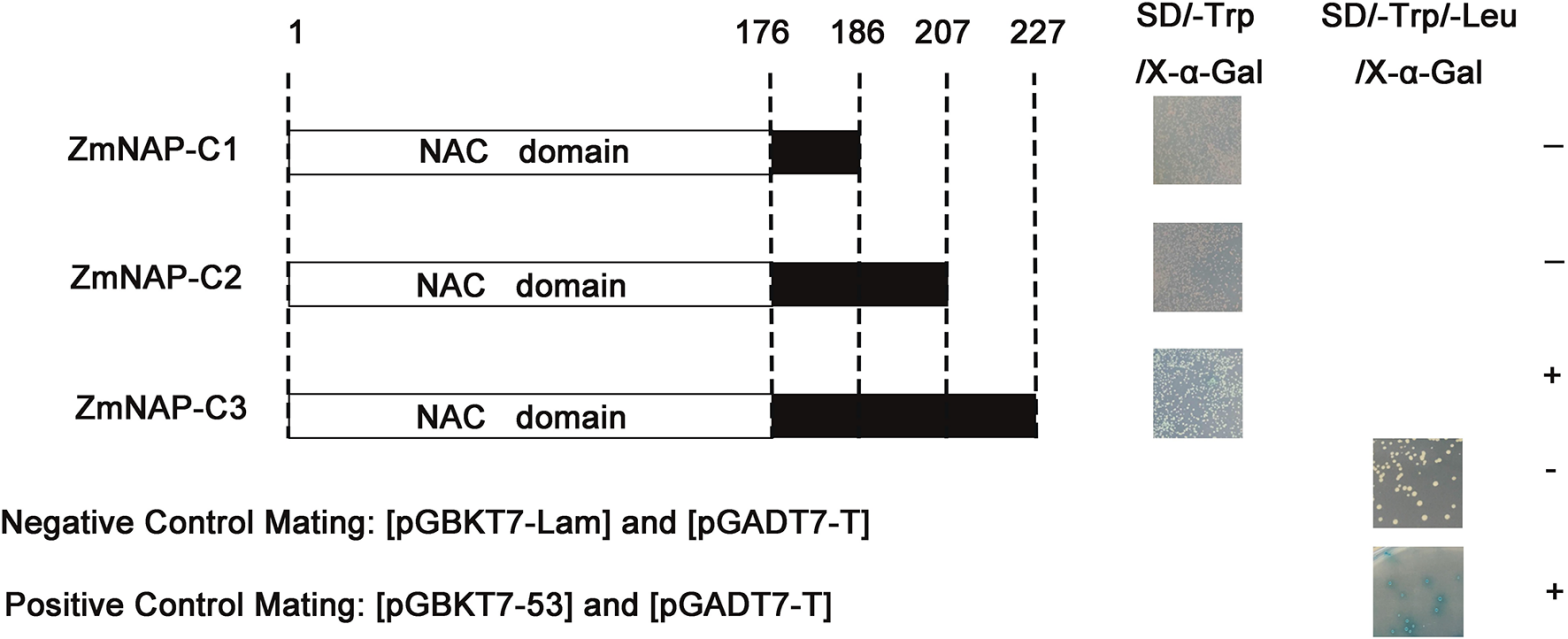
Screening and identification of ZmNAP interacting proteins in Y2HGlod yeast. Three truncations of ZmNAP were ligated into the pGBKT7 vector. Recombinant vectors were transformed into Y2HGold and selected on SD/-Trp/X-α-Gal and SD/-Trp/-Leu/X-α-Gal. The pGBKT7-Lam and pGADT7-T were used as the negative control. The pGBKT7-53 and pGADT7-T were used as the positive control. Experiments were independently repeated at least three times.

**Figure 12:**
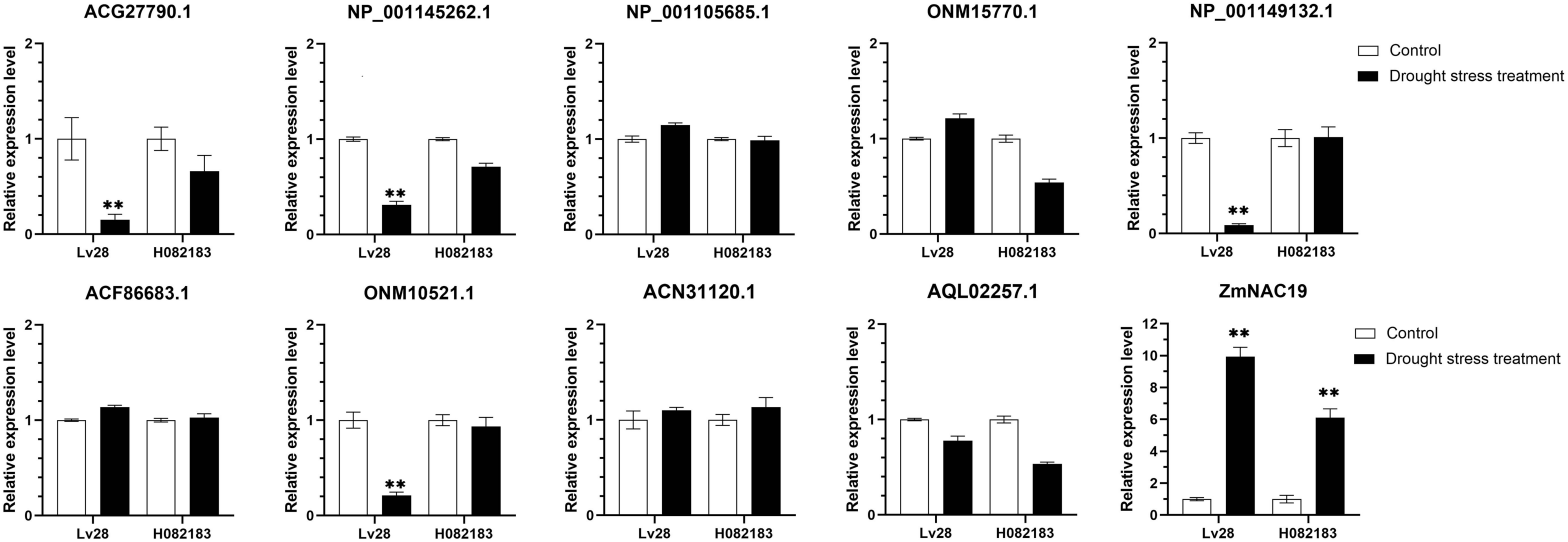
Expression analysis of ZmNAP interacting protein coding genes under drought stress in Lv28 and H082183. Maize *ZmGAPDH (NM_001111943)* was used as the internal reference gene. Highly significant differences (P < 0.01) were represented by **.

NAC TFs perform multiple functions by interacting with other proteins. ZmNAP, which is differently expressed in two maize inbred lines with contrasting drought tolerance, could significantly enhance drought stress tolerance. We further identified the interacting proteins of ZmNAP by yeast two-hybrid screening (**Figure 11**). After screening and rotation verification, 11 proteins were identified that could potentially interact with ZmNAP (**Supplementary Table 4**). Gene function annotations identified that the interacting protein NP_001105685.1 was associated with the synthesis of cysteine proteinase; cysteine proteases have been reported to participate in numerous developmental processes and abiotic stress (Koizumi et al., 1993; McLellan et al., 2009). AQL02257.1 is a key enzyme synthesis gene of glycolysis and involved in the process of sugar metabolism. The interacting protein NP_001145262.1 has been reported to be involved in chloroplast development. The expression of the interacting protein-coding genes in Lv28 and H082183 indicated that *ACG27790.1*, *NP 001145262.1*, *NP 001149132.1*, and *ONM10521.1* were downregulated in Lv28 under drought stress conditions, whereas no significant differences could be detected in H082183 (**Figure 12**). Notably, ZmNAC19, which is one interacting protein of ZmNAP, was significantly induced by drought treatment in both Lv28 and H082183. It is speculated that ZmNAP may form a heterodimer with ZmNAC19 to increase drought resistance.

## Discussions

NAC transcriptional factors constitute one of the largest TF families in plants and play vital roles in plant development and abiotic stress resistance (Olsen et al., 2005; Jensen and Skriver, 2014). So far, at least 117, 151, and 260 NAC genes have been identified in *Arabidopsis*, rice, and wheat (Nuruzzaman et al., 2010; Puranik et al., 2012; Mao et al., 2020). However, the characterization of the drought tolerance function of maize NACs has been limited to only a few members.

Genome-wide gene expression analysis by RNA-Seq provides a powerful method to mine drought tolerance–related genes in maize. Numerous genes responsive to drought may show a genotypic differential expression between H082183 and Lv28 with contrasting drought tolerance. In this study, we compared the transcriptional sequencing data of the NAC TFs of the two inbred lines under well-watered conditions and natural soil drought stress, to screen the NAC family genes that were specifically differentially expressed between the two genotypes under drought conditions. There were 32 NAC genes differentially expressed between the two genotypes under at least one drought stress condition. Among them, three NAC genes encoded GRMZM2G031200, GRMZM2G430849, and GRMZM2G475014 were differentially expressed in both MD and SD. The relative higher expression of these NAC genes in the drought-tolerant inbred line H082183 suggests that they may participate in drought resistance and adaptation in maize. There were 28 NAC genes upregulated in the drought-tolerant line H082183 under at least one drought stress condition, including three well-known maize drought stress–responsive genes, *ZmSNAC1*, *ZmNAC55*, and *ZmNAC111*, which have been reported to significantly improve plant drought tolerance (Lu et al., 2012; Mao et al., 2015; Mao et al., 2016).

By referring to the Plant Transcription Factor Database, 152 non-redundant NAC protein sequences were screened for evolution analysis. The maize NAC family proteins were grouped into 13 distinct subfamilies. A series of drought stress–responsive maize NAC members such as ZmSNAC1, ZmNAC55, ZmNAC111, ZmNAC84, ZmNAC33, and ZmSNAC13 fell into OsNAC3, ONAC3, NEO, and ATAF subfamilies (Lu et al., 2012; Mao et al., 2015; Mao et al., 2016; Zhu et al., 2016; Liu et al., 2019; Luo et al., 2022). In the present study, the RT-qPCR assay also confirmed that *GRMZM2G180328*, *GRMZM2G127379*, *GRMZM2G430849*, *GRMZM2G163251*, *GRMZM2G181605*, and *GRMZM2G081939* belonging to these subfamilies were significantly induced by drought stress in maize B73 seedlings.

Numerous reports have indicated that the overexpression of stress-related NAC genes could improve the stress resistance of transgenic plants. For instance, the overexpression of rice *OsNAC2* improved transgenic plants’ sensitivity to high salt and drought stress (Yu et al., 2021). The overexpression of *ZmNAC55* enhanced drought stress tolerance in transgenic *Arabidopsis* (Mao et al., 2016). Maize *ZmNAC33* overexpressing *Arabidopsis* performed improved osmotic stress tolerance and ABA sensitivity at the seed germination stage and enhanced drought stress tolerance (Liu et al., 2019). In this study, five NAC genes *ZmNAP*, *ZmNAC19*, *ZmNAC4*, *ZmJUB1*, and *ZmNAC87*, which were induced more than twofold by MD or SD treatments, were selected according to the transcriptome sequencing data. *ZmNAP-*, *ZmNAC19-*, *ZmNAC4-*, *ZmJUB1-*, and *ZmNAC87*-overexpressing lines significantly increased the germination sensitivity to various abiotic stress and ABA treatments, and the survival rates of transgenic seedlings were significantly higher than that of the WT plants under drought stress treatment. This may be inferred that ZmNAP, ZmNAC19, ZmNAC4, ZmJUB1, and ZmNAC87 play critical roles in the plant response to drought stress. ZmNAC4, which was designed as ZmNAC13 in the previous study, also resulted in enhanced drought stress tolerances in *Arabidopsis* (Luo et al., 2021).

Generally, phylogenetically clustered genes share a higher sequence identity and have similar biological functions. Multiple sequence alignment indicated that ZmNAP, ZmNAC19, ZmNAC4, ZmJUB1, and ZmNAC87 were confirmed to possess a typical NAC domain and a transactivation domain located at the C terminus (**Supplementary Figure 2**), which had the highest sequence identity to AtNAP, SNAC1, OsNAC4, JUB1, and ANAC087. In *Arabidopsis*, AtNAP and ANAC087 were induced by ABA and abiotic stress and promoted leaf senescence in *Arabidopsis* (Guo and Gan, 2006; Huysmans et al., 2018). JUB1 was a multifunctional member of the NAC TFs that showed to be involved in responses to abiotic stresses (Ebrahimian-Motlagh et al., 2017). OsNAC4 led to hypersensitive response (HR) programmed cell death to participate in plant immune responses (Kaneda et al., 2009). *SNAC1* was a drought-inducible gene in rice and significantly improved drought and salt stress tolerance (Hu et al., 2006). In the present study, ZmNAP-, ZmNAC19-, ZmNAC4-, ZmJUB1-, and ZmNAC87-overexpressing *Arabidopsis* exhibited a significant enhancement of drought stress tolerance. These results inferred that ZmNAP, ZmNAC19, ZmNAC4, ZmJUB1, and ZmNAC87 may play important roles in drought response regulatory networks. Furthermore, yeast two-hybrid screening suggested that ZmNAP interacts with ZmNAC19 in yeast. The dimerization of the NAC domain is common and can function in modulating the DNA-binding specificity (Müller, 2001). ZmNAP may form a heterodimer with ZmNAC19 to participate in the drought tolerance regulatory network.

ABA plays an important role in abiotic stress responses, regulating various stages of plant development, such as seed maturation, dormancy, organ abscission, and leaf senescence. NAC proteins regulate biotic and abiotic stress responses through ABA-dependent and -independent signaling pathways. *ZmNAP*, *ZmNAC19*, *ZmNAC4*, *ZmJUB1*, and *ZmNAC87* were induced by drought stress, dehydration, PEG and ABA treatments. Furthermore, the promoters of these five NAC genes contain several ABA responsive elements (ABREs). Our data show that ZmNAP, ZmNAC19, ZmNAC4, ZmJUB1, and ZmNAC87 may respond to abiotic stresses in ABA-dependent signaling pathways.

In conclusion, in this study, five maize NAC TFs, ZmNAP, ZmNAC19, ZmNAC4, ZmJUB1, and ZmNAC87, were identified according to the transcriptome analysis. The full-length CDSs and C-terminal regions of these five NAC proteins performed the transactivation activity. Relative expression analysis revealed that *ZmNAP*, *ZmNAC19*, *ZmNAC4*, *ZmJUB1*, and *ZmNAC87* were strongly induced by drought stress, dehydration, and PEG and ABA treatments. The overexpression of *ZmNAP*, *ZmNAC19*, *ZmNAC4*, *ZmJUB1*, and *ZmNAC87* in *Arabidopsis* increased sensitivity to ABA, NaCl, and D-mannitol at the germination stage, and the drought tolerance of transgenic lines was significantly improved compared with WT. Moreover, ZmNAP may physically interact with 11 proteins including ZmNAC19 to activate the drought stress response. These results indicated that ZmNAP, ZmNAC19, ZmNAC4, ZmJUB1, and ZmNAC87 may activate the drought stress response *via* ABA-dependent signaling pathways. The data may be useful for improving maize drought stress tolerance through molecular breeding programs.

## Data availability statement

The datasets presented in this study can be found in online repositories. The names of the repository/repositories and accession number(s) can be found in the article/**Supplementary Material**.

## Author contributions

The authors ND, YZ, and WW contributed equally to this work. All authors contributed to the article and approved the submitted version.
