# Supplementary material for: Transcriptome analysis in contrasting maize inbred lines and functional analysis of five maize NAC genes under drought stress treatment
Source: Front Plant Sci. 2023 Jan 19;13:1097719. doi: 10.3389/fpls.2022.1097719 (PMC9892906; doi:10.3389/fpls.2022.1097719)
Supplement: Supplementary file 1 [file Table_1.docx]

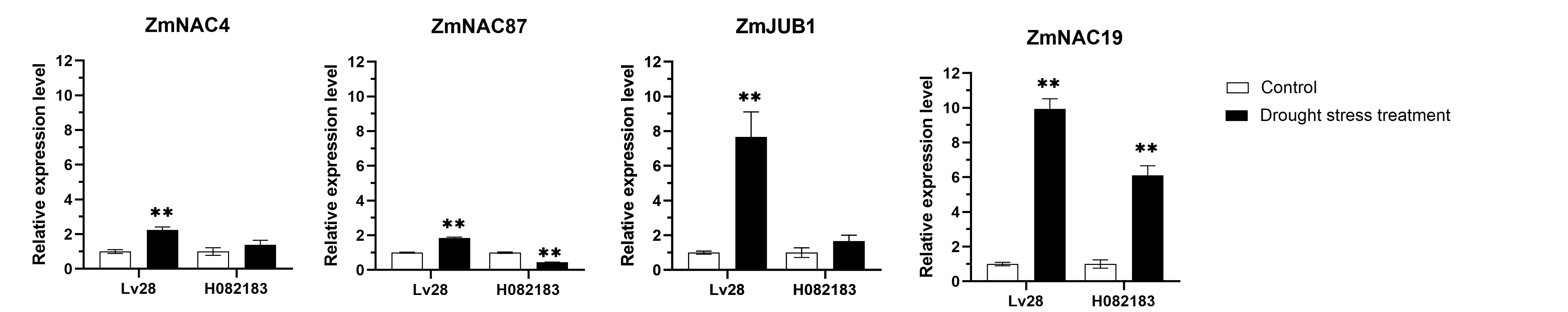


Supplementary Figure 1. Expression levels of NAC genes under drought stress.


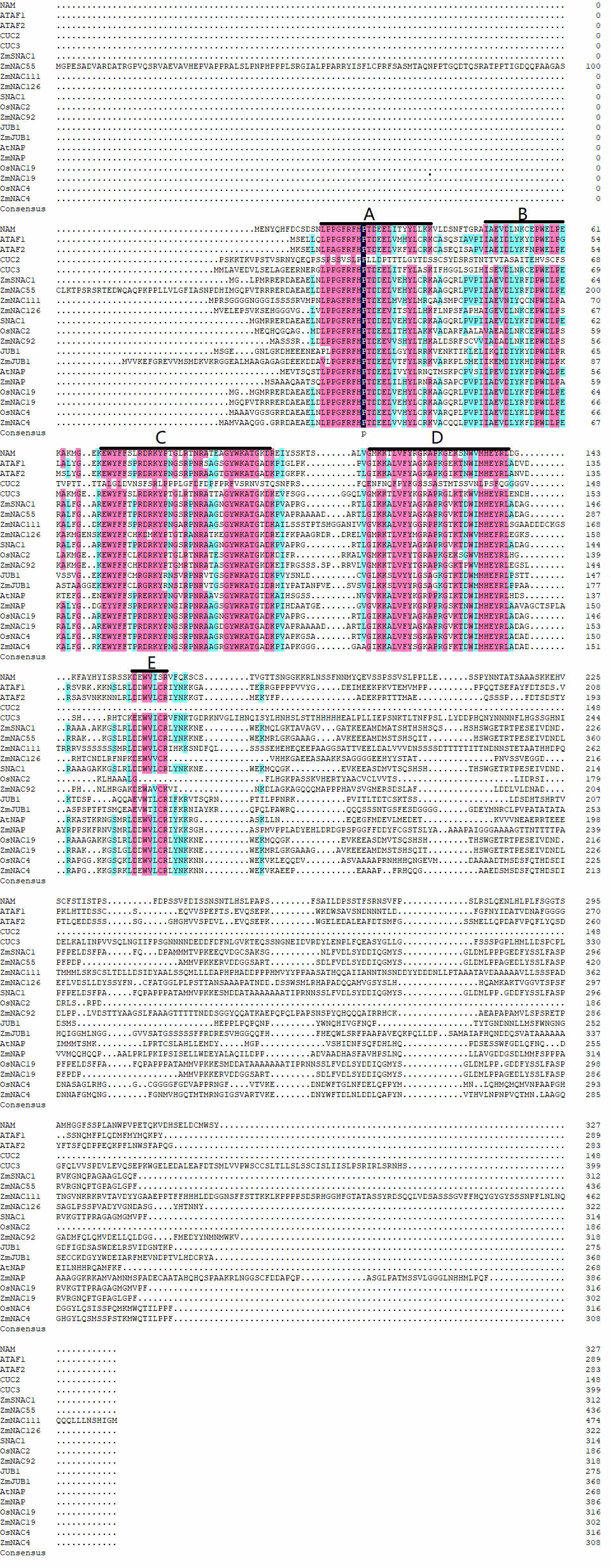


Supplementary Figure 2. Comparison of the amino acid sequences of NAC proteins.
